# At-Home Morning Bright Light Treatment for Chronic Nociplastic Pain: Protocol for a Randomized Clinical Trial

**DOI:** 10.2196/75060

**Published:** 2025-10-13

**Authors:** Helen J Burgess, Allie A Rodgers, Kiley A McNeil, Jackson Mott, Agnes Fejer, Tori Dereski, Muneer Rizvydeen, Kimberly T Sibille, Hyungjin Myra Kim, Cherie Cofield, John W Burns, Sana Shaikh, Afton L Hassett

**Affiliations:** 1 Sleep and Circadian Research Laboratory, Department of Psychiatry University of Michigan Ann Arbor, MI United States; 2 Department of Anesthesiology University of Michigan Ann Arbor, MI United States; 3 Department of Physical Medicine & Rehabilitation University of Florida Gainesville, FL United States; 4 Pain Research and Intervention Center of Excellence University of Florida Gainesville, FL United States; 5 Department of Anesthesiology, Division of Pain Medicine University of Florida Gainesville, FL United States; 6 Department of Biostatistics University of Michigan Ann Arbor, MI United States; 7 School of Nursing Emory University Atlanta, GA United States; 8 Department of Psychiatry and Behavioral Sciences Rush University Medical Center Chicago, IL United States

**Keywords:** fibromyalgia, light, mood, nociplastic, pain, sleep

## Abstract

**Background:**

Fibromyalgia, the quintessential nociplastic pain condition, affects more than 20 million Americans and results in significant disability, lost productivity, and poor quality of life, with profound individual and societal cost. As pharmacological treatment approaches offer only modest benefits and result in a high rate of discontinuation due to adverse effects, nonpharmacological interventions such as physical therapy, exercise, and cognitive behavioral therapy are recommended. However, cost and access to these treatments can create barriers to care, and engagement can be problematic. Morning bright light treatment is a promising option for improving fibromyalgia symptoms, with early studies indicating clinically meaningful improvements in fibromyalgia symptoms.

**Objective:**

This study aims to prospectively examine the potential benefits and active elements of morning bright light treatment and sleep timing stabilization for individuals with fibromyalgia in the largest randomized controlled trial to date.

**Methods:**

We will recruit 390 adults who meet diagnostic criteria for fibromyalgia and report at least mild symptoms. Participants will be randomized to one of three groups: 4 weeks of morning bright light treatment (1 hour per day, using a commercially available Re-timer device), 4 weeks of sleep timing stabilization alone (a component of morning bright light treatment, some benefit anticipated), or 4 weeks of treatment as usual, with equivalent study contact. Patient-reported outcomes of function and pain will be assessed before and after treatment, with mood, sleep quality, and morningness-eveningness examined as potential mediators of treatment effects. Social determinants of health risk will be examined as a potential moderator influencing baseline symptoms, treatment engagement, and treatment response.

**Results:**

Data collection began in September 2024 and is projected to end in March 2029.

**Conclusions:**

Morning bright light treatment is well-positioned to be an effective nonpharmacological treatment for fibromyalgia with minimal side effects. The study findings will provide important insights relevant to the development of morning bright light treatment as an accessible treatment for chronic nociplastic pain.

**Trial Registration:**

ClinicalTrials.gov NCT06567886; https://clinicaltrials.gov/study/NCT06567886

**International Registered Report Identifier (IRRID):**

DERR1-10.2196/75060

## Introduction

Chronic nociplastic pain includes conditions such as irritable bowel syndrome, interstitial cystitis, and the quintessential nociplastic pain condition, fibromyalgia, which alone affects more than 20 million Americans [1]. While the mechanisms underlying nociplastic pain are not completely understood, central nervous system–mediated amplification of pain and other sensory input, along with altered pain modulation, play a major role [2]. In addition to pain, mood and sleep disturbances, fatigue, and cognitive symptoms are common [3,4]. Resultant disability, lost productivity, and poor quality of life have profound costs at the individual and societal level [5]. Underserved individuals experience these impacts more profoundly [6] as symptom burden is heavily influenced by demographic risk factors, including social determinants of health (SDOH: eg, education, income, medical insurance, and community resources) [7-10].

While a pharmacological approach has traditionally been the first line of treatment for fibromyalgia, drug therapies offer only small benefits [11-14] and result in a high rate of discontinuation due to adverse effects [11]. Newer national guidelines suggest that the initial approach to the treatment of fibromyalgia consists of non-pharmacological interventions [15-17]. Physical therapy, exercise, and psychological therapies such as cognitive behavioral therapy (CBT) have demonstrated efficacy but can be difficult to access in terms of cost and availability [18-20], and engagement can be problematic [21]. Indeed, individuals with chronic pain are most adversely impacted by SDOH, have the least access and ability to consistently engage in nonpharmacological therapies [7]. Thus, a need exists to develop novel adjunctive approaches to manage fibromyalgia symptoms that have optimal treatment effects, minimal side effects, and are easy to implement and readily accessible for all.

**Figure 1:**
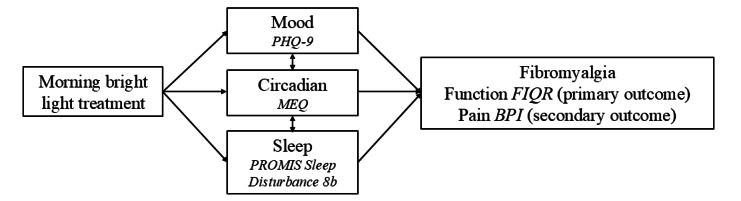
A conceptual model illustrating that morning bright light treatment, which is known to improve mood and sleep and to shift circadian timing earlier, has the potential to improve fibromyalgia. The measure used to assess each domain is shown in italics: Patient Health Questionnaire (PHQ-9), Morningness-Eveningness Questionnaire (MEQ), PROMIS Sleep Disturbance 8b, Fibromyalgia Impact Questionnaire–Revised (FIQR), and Brief Pain Inventory (BPI).

Morning bright light treatment is a promising option for improving fibromyalgia symptoms. Meta-analyses have confirmed that morning light treatment improves mood (reduces nonseasonal depression) with medium effect sizes similar to those observed with pharmacological antidepressants [22,23]. Similarly, a meta-analysis indicated that morning light treatment improves sleep with medium effect sizes [24]. Improvements in mood and sleep are well-recognized as contributing to improvements in chronic pain [25,26]. Morning bright light treatment also advances circadian timing (shifts circadian timing earlier toward “morningness” [27]). As later circadian timing (“eveningness”) is associated with worse fibromyalgia pain [28,29], circadian phase advances associated with morning bright light treatment may help reduce symptoms (see Figure 1). In addition, emerging research in rodent models suggests green light exposure to be antinociceptive [30,31]. Importantly, while light treatment is associated with some side effects (headache, eyestrain, nausea, and agitation [32]), these symptoms often spontaneously remit [32,33], and patients rarely discontinue treatment due to these side effects [33]. Furthermore, light devices with a UV filter are considered safe with no changes in ophthalmologic examinations observed after 6 years of daily use (in fall and winter months) [34].

To date, only 3 studies have tested light treatment in people with fibromyalgia. In our first study, we tested 6 days of either a 1-hour evening or 1-hour morning bright light treatment in 10 participants with fibromyalgia [35]. Only the morning bright light treatment led to clinically meaningful improvements in function (>14% reduction in baseline Fibromyalgia Impact Questionnaire—Revised [FIQR] score [36]). In this study, we travelled to participants’ homes to set up white light boxes, which was burdensome for participants and staff. This experience led us to switch to a novel commercially wearable light device (Re-timer), which permits ambulation while receiving light from green LEDs positioned below the eyes. Our second study was a randomized clinical trial that tested 4 weeks of a 1-hour daily morning light treatment (bright or dim) using the Re-timer in 60 people with fibromyalgia [37]. We found that both groups responded to the morning light treatment, with clinically meaningful improvements in the FIQR (mean decrease of 11.2, *P*=.003). The findings were similar to those seen after exercise and were double those seen with CBT or analgesics (meta-analysis [38]). A third study by Martin et al [39] tested 7-9 weeks of a 1-2 hour daily white versus green light treatment in 21 people with fibromyalgia. The light was generated from LED strips, and the time of day of light exposure was not reported. They found that only the green LED light led to significant reductions in pain intensity, and quality of life and self-reported function also improved. Overall, morning bright light treatment is well-positioned to be a potentially accessible and scalable nonpharmacological treatment for fibromyalgia with minimal side effects. Furthermore, light treatment devices are commercially available, and light treatment can be self-administered at home, allowing easy access and dissemination once efficacy is established.

To further examine the potential benefits and active elements of morning bright light treatment for people with fibromyalgia, we will conduct a larger parallel groups randomized controlled trial with 3 conditions: 4 weeks of 1 hour/day green morning bright light treatment (Re-timer), a sleep timing stabilization alone treatment (a component of morning bright light treatment, some benefit anticipated [40-42]) and treatment as usual (treatment as usual [TAU], a control group to control for attention and time in study effects). We hypothesize that (1) participants who receive morning bright light treatment will show greater improvements in function and pain; (2) that SDOH risk will influence baseline symptom burden, treatment adherence (engagement), and treatment response; and (3) that treatment response may be partially mediated by improvements in depressive symptoms and sleep, and a shift toward morningness. The study findings will provide important insights relevant to the development of morning bright light treatment as an accessible treatment for chronic nociplastic pain.

## Methods

### Study Design

**Figure 2:**
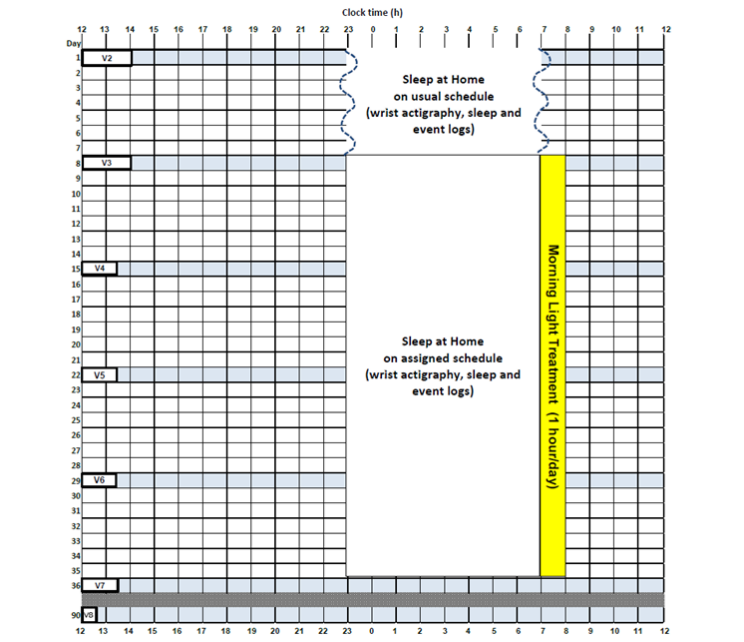
A diagram of the 5-week study protocol with a 3-month follow-up for a participant with an average sleep schedule of 11 PM to 7 AM who is assigned to the morning light treatment. At visit 2 (V2), participants are instructed on how to wear a wrist actigraphy monitor and complete daily logs while sleeping ad lib at home. A week later, at visit 3 (pretreatment, V3), outcome measures are collected and participants are informed of their randomization to morning light treatment, sleep stabilization, or treatment as usual (TAU). During weekly visits (V4, V5, V6, and V7) participants’ adherence to the light treatment and/or sleep schedule are checked (if assigned to morning light treatment or sleep stabilization), and daily logs and side effects are reviewed (all groups). Outcome measures are collected again during visit 7 (post-treatment, V7) and 3 months later during visit 8 (V8). The sleep stabilization study protocol looks similar to the morning light treatment protocol except there is no morning light treatment. The TAU study protocol looks similar to the morning light treatment protocol except the ad lib sleep during the first week of the study continues throughout the 5-week study protocol. The anticipated duration of each study visit is shown.

The FM Sleep A to ZZZ Study is a prospective single-center randomized clinical trial with a 3-arm parallel groups design comparing 4 weeks of morning light treatment, sleep stabilization, and TAU in patients with fibromyalgia. The TAU group will be presented to participants as a study of sleep monitoring to minimize group differences in treatment expectations. A sample diagram of the study protocol is shown in Figure 2. There are weekly study visits during the 5-week protocol, with outcome variables assessed after a week of sleep monitoring at pre-treatment (visit 3), 4 weeks later at posttreatment (visit 7), and then a follow-up 3 months after posttreatment (visit 8). All study visits will occur remotely via Zoom (Zoom Video Communications).

### Ethical Considerations

The University of Michigan Medical School Institutional Review Board approved the research study before launch (HUM00240590), and all participants are required to provide a written informed consent document before study participation. The informed consent document outlines that the participants can withdraw from the study at any time. All research staff are required to maintain training in human participants’ research, including how to maintain privacy and confidentiality. As part of the study, personal identifying information is only collected on an as-needed basis and stored in a secure REDCap (Research Electronic Data Capture; Vanderbilt University) database, or in written progress notes that are stored under lock and key in the Sleep and Circadian Research Laboratory. The clinical trial has been registered on ClinicalTrials.gov (NCT06567886). Once participants are accepted into the study, they are compensated after they complete each study visit: US $50 after Visit 2 and 8, US $100 after Visit 3, 4, 5, 6, and 7. Participants also receive a US $50 study completion bonus, as well as US $50 when the study supplies are returned, for a total payment of up to US $700.

### Setting

Study participants will be recruited by the University of Michigan’s Back & Pain Center using various resources of the University of Michigan’s health system. The University of Michigan’s Back & Pain Center is a large tertiary pain clinic that assesses around 5000 patients every year. A clinical pain research team is embedded with the clinic and has established the APOLO registry [43]. This large registry provides an ample means for recruitment of fibromyalgia patients, as most of the patients in the registry have agreed to be contacted for research studies. In addition, with Institutional Review Board approval, potentially eligible participants will be identified through a review of electronic medical records when available, and the study will be advertised on the University of Michigan Research Participant Registry [44]. Finally, the services provided by the Publicly Engaged Research Core within the Chronic Pain and Fatigue Research Center at the University of Michigan will also be used to prioritize the recruitment and enrollment of high SDOH risk participants. All study visits will occur with staff located at the Sleep and Circadian Research Laboratory in the Department of Psychiatry at the University of Michigan.

### Study Population

We aim to enroll 390 individuals, aged 18 years or older, who meet the 2016 revised diagnostic criteria for fibromyalgia [45] (see Textbox 1). We anticipate ~70% of the sample will be female based on our previous work [46,47]. To ensure participants have at least mild symptoms, a FIQR score of ≥31 will also be required [48]. We have chosen inclusion and exclusion criteria to permit as many people with fibromyalgia as possible to participate safely, while maximizing the generalizability of findings. For example, prescribed hypnotics, over-the-counter sleep aids, antiepileptics, antidepressants, opioid analgesics, and muscle relaxants will be permitted as their use is common in fibromyalgia, but these permitted medications will need to be stable for 30 days before and during the study. Participants will be able to continue psychological therapy, physical therapy, and exercise if the treatment was started 30 days before enrollment and continues during the study period. Participants must have internet access (a cell phone can be provided for the study if required) and a private space for the remote study visits. Participation will be scheduled ≥1 month from night work, travel outside the local time zone, and other research participation, and will be scheduled during a period with minimal special events (eg, weddings, concerts, examinations).

**Inclusion and exclusion criteria.**

Inclusion criteria:

1. Age ≥18 years old
2. Meets 2016 revised diagnostic criteria for fibromyalgia:
  - Symptoms present ≥3 months.
  - Widespread Pain Index (WPI) ≥7 & Symptom Severity (SS) ≥5 or WPI 4-6 and SS ≥9.
  - Pain reported in at least 4 of 5 body regions.
3. Fibromyalgia Impact Questionnaire–Revised (FIQR) score ≥31.
4. Fluent in English.
5. Access to the internet (study cell phone provided if required).
6. Private space for remote study visits.

Exclusion criteria:

1. Health:
  - Other significant chronic physical disease (eg, autoimmune disorders, uncontrolled diabetes, advanced liver disease, cancer, kidney failure, uncontrolled cardiovascular disease, seizures, light-triggered migraines).
  - Has a pacemaker or defibrillator (potential interference from Fitbit device).
  - Retinal pathology, cataracts, glaucoma, and colorblindness.
  - Lifetime history of psychotic or bipolar disorders.
  - Suicidality in the past 6 months
  - Alcohol or substance use disorder in the past 3 months (cannabis use ≤1/week ok).
  - High risk for or diagnosed with narcolepsy.
  - Severe hearing problem or significant cognitive impairment.
  - Pregnant, trying to get pregnant, or breastfeeding.
  - Has a child or pet at home that disturbs sleep often.
  - Pending medical leave application at work, pending legal case or litigation.
  - Working shiftwork that affects sleep.
  - Recent (<1 month) travel outside the local time zone.
  - Participating in another research study.
2. Medications:
  - Taking photosensitizing medications to blue-green light.
  - Unstable medication use 30 days before or during the study.
3. Light treatment:
  - Average wake time is before 5 AM.
  - Unable to fit 1 hour of light treatment into morning routine.
  - Previous use of light treatment in the past 6 months.

### Procedures and Assessments

Potential participants will be prescreened for major inclusion and exclusion criteria using a telephone interview conducted by staff in the Back & Pain Center in the Department of Anesthesiology at the University of Michigan. If eligible, participants will then complete visit 1 (eligibility visit), which includes obtaining written informed consent and collection of self-report measures related to clinical history, demographics, and health information to determine further eligibility. Medications will be reviewed with available medical records and confirmed with each participant for accuracy. Height, weight, and visual impairment, including colorblindness, will also be collected via self-report due to the remote nature of the study (see schedule of assessments in Table 1).

**Table 1:** Schedule of measure administration.

Measures			Screening					Study visits													3-month follow-up
			Phone interview		Visit 1		Visit 2			Visit 3		Visit 4		Visit 5		Visit 6		Visit 7		Visit 8	
**Screening and baseline measures**																					
	Informed consent, eligibility confirmation			✓																	
	Demographics	✓		✓																	
	Height and weight (self-report)			✓																	
	Medical history, medications	✓		✓																	
	Social determinants of health (SDOH) risk score			✓																	
	Experiences of discrimination (EOD)					✓															
	Multidimensional Scale of Perceived Social Support (MSPSS)					✓															
	CDC Social Vulnerability Index (SVI)					✓															
	Restless legs (CHQ^a^ and sIRLS^b^)					✓															
	Sleep apnea (STOP-BANG)					✓															
	Positive and Negative Affect Schedule (PANAS)					✓															
**Self-report measures**																					
	FIQR^c^ (primary outcome)	✓							✓								✓		✓		
	BPI^d^ (secondary outcome)								✓								✓		✓		
	PHQ-9^e^								✓								✓				
	PROMIS^f^ Sleep Disturbance 8b								✓								✓				
	Morningness-Eveningness Questionnaire (MEQ)								✓								✓				
	Daily logs (sleep and events)					✓			✓		✓		✓		✓		✓				
	Treatment expectation								✓												
	Treatment satisfaction																✓				
**Objective measures**																					
	Wrist actigraphy					✓			✓		✓		✓		✓		✓				
**Safety Measures**																					
	C-SSRS^g^ (past 6 months)			✓																	
	C-SSRS (since last visit)					✓			✓		✓		✓		✓		✓		✓		
	BDI^h^					✓			✓		✓		✓		✓		✓				
	SAFTEE^i^										✓		✓		✓		✓				

^a^CHQ: Cambridge Hopkins Questionnaire.

^b^sIRLS: self-administered International Restless Legs Syndrome Severity Rating Scale.

^c^FIQR: Fibromyalgia Impact Questionnaire—Revised.

^d^BPI: Brief Pain Inventory.

^e^PHQ-9: Patient Health Questionnaire—9.

^f^PROMIS: Patient-Reported Outcomes Measurement Information System.

^g^C-SSRS: Columbia Suicide Severity Rating Scale.

^h^BDI: Beck Depression Inventory.

^i^SAFTEE: systematic assessment for treatment emergent events.

Eligible participants will be enrolled in the study at visit 2, during which questionnaires to characterize the sample will be completed (Table 1). Before visit 2, participants will be randomized to the morning light treatment, sleep stabilization treatment, or TAU group, and will be mailed the appropriate study supplies (see below for more details). All participants will receive a Fitbit Charge 5 or 6 to wear on their nondominant wrist to track their sleep and activity throughout the 5-week study protocol, along with daily logs to report their subjective experience of sleep and caffeine, alcohol, and medication use. A week later, at visit 3, pre-treatment outcome assessments will be administered, and treatment expectations will be assessed. The randomly assigned treatment condition will then begin the morning after visit 3. Thereafter, there will be weekly visits (visits 4, 5, and 6) to review each participant’s adherence to instructions (sleep timing alone or paired with the use of the Re-timer, depending on treatment group) and to systematically assess side effects (all groups). At visit 7, posttreatment outcome measures will be administered, and study satisfaction will be assessed. Participants will be required to abstain from alcohol and cannabis use within 24 hours of visits, with reminders the day before each visit.

### Randomization and Blinding

Participants will be randomized to either 4 weeks of morning light treatment (1 hour per day), 4 weeks of sleep timing stabilization alone, or 4 weeks of TAU in a 1:1:1 ratio. Randomization will use a minimization approach to reduce imbalances in important variables, including FIQR score (31-65 vs 66-100 to reflect the divide between mild to moderate symptoms vs severe symptoms), age (18-44 vs ≥45 years to reflect that more circadian disruption is observed in younger participants and the prevalence of FM is higher ≥45 years), biological sex (male or female), and SDOH risk index (0-2 vs 3-6, explained below). Study research assistants assessing outcomes at study visits 3 and 7 will be blinded to treatment assignment and instructed not to discuss any aspect of the treatment with participants. The participants will also be instructed not to discuss the study treatment with the blinded research staff. Otherwise, unblinded study staff will meet with participants weekly to download data from the wrist monitor and Re-timer device (in the morning light treatment group), provide feedback on adherence, and assess treatment side effects.

### Intervention: Morning Light Treatment

The morning light treatment will be self-administered at home using the commercially available wearable Re-timer light therapy glasses, which emit a green light and are designed to optimize therapeutic wavelength (~500 nm, 230 µW/m^2^, and 500 lux) by being close to the peak sensitivity of the circadian photoreceptors (~480 nm) [49,50]. The Re-timer device can be worn over glasses and does not interfere with ambulation, vision, reading, or computer work. Participants will be instructed to start the light treatment the morning after visit 3, immediately following their assigned wake time. Their assigned wake time will be their average final wake time as determined during the baseline week, or up to an hour earlier than their average wake time, to allow time for light treatment [37,51,52]. If the participant’s assigned wake time is advanced from their average wake time, then their bedtime will also be shifted earlier to avoid sleep deprivation. The earliest start time for light treatment is 6 AM. The Re-timer device automatically turns off after 1 hour. Participants will complete the 1-hour light treatment sessions for 28 consecutive days at the same time each day. Participants will also be instructed not to sleep or meditate (closing eyes) within five hours after their assigned light treatment start time, to avoid creating a dark pulse that can counteract the effect of the light.

Adherence will be assessed via light and actigraphy data measured by a monitor (1-minute epochs, Actlumus, Condor Instruments) attached to the Re-timer, which will be reviewed with participants at the weekly study visits by unblinded staff. Research staff will contact all participants via phone call or text 10 minutes after their assigned light treatment start time each morning to ensure that they have started the light treatment. Participants will also be provided an alarm clock set to their assigned wake time to promote adherence.

### Intervention: Sleep Stabilization

Participants will be instructed to start following an assigned sleep schedule the morning after visit 3. Similar to the light treatment intervention, the assigned wake time will be their average final wake time as determined during the baseline week, or up to 1 hour earlier than their average wake time. If the participant’s assigned wake time is advanced from their average wake time, then their bedtime will also be shifted earlier to avoid sleep deprivation. Participants will also be instructed not to sleep or meditate (closing eyes) within 5 hours after their assigned wake time, to avoid creating a morning dark pulse that may shift circadian timing.

Adherence will be assessed via actigraphy data measured by the Fitbit Charge 5 or 6, which will be reviewed with participants at the weekly study visits by unblinded staff. Research staff will contact all participants via phone call or text 10 minutes after their assigned wake time each morning to ensure that they are awake and out of bed. Participants will also be provided an alarm clock set to their assigned wake time to promote adherence.

### Comparator: Treatment as Usual

Participants assigned to TAU will be instructed at visit 3 to follow their usual ad lib sleep schedule, as they did during the baseline week. Research staff will contact these participants via phone call or text each day to encourage them to complete their daily logs. This contact, together with the weekly study visits, will ensure equivalent study contact and attention between the morning light treatment group and the TAU group. The TAU participants' wrist actigraphy data will also be reviewed weekly during the Zoom visits, with feedback on following the study procedures.

### Outcome Measures

Study outcomes will be assessed in each group at pretreatment (visit 3), posttreatment (visit 7), and then at a follow-up 3 months after posttreatment (visit 8) to evaluate the duration of any changes in each treatment condition. The study assessment schedule is shown in Table 1.

### Self-Reported Outcomes

The primary outcome measure is the FIQR [48]. The FIQR consists of 21 items that assess function, overall impact, and symptoms. Total scores range from 0 to 100, with higher scores representing worse functional status. The secondary outcome measure is the Brief Pain Inventory (BPI), which assesses for the presence of pain, pain intensity, and functional interference from pain [53]. The BPI consists of 2 scores (pain severity and pain interference) with higher scores indicating more pain or greater pain interference. The BPI has been validated for fibromyalgia specifically [54,55].

Three additional outcome measures will be explored as potential mediators. The Patient Health Questionnaire-9 (PHQ-9) is a reliable measure of the severity of depressive symptoms [56]. The PHQ-9 consists of 9 items, and higher scores reflect higher levels of depressive symptoms. The Patient-Reported Outcomes Measurement Information System (PROMIS) Sleep Disturbance Short-Form 8b is a reliable measure of sleep quality [57]. Higher scores on the PROMIS Sleep Disturbance Short-Form 8b reflect worse sleep quality. The Morningness-Eveningness Questionnaire (MEQ) [58] will be used as a proxy marker of circadian timing. The MEQ score correlates well with the gold standard circadian phase marker, the dim light melatonin onset [59], and significantly increases towards more “morningness” after 13-28 days of a morning light treatment [60]. This will allow us to confirm an expected phase advance or earlier shift in circadian timing in response to morning light treatment.

### Social Determinants of Health Measures

At visit 1, an SDOH index score will be derived from individual and community-level data consistent with previous publications [61,62]. Individual-level data collected will include education, household income (and number of individuals in household to assess poverty level), marital or partner status, employment, and medical insurance status. Community-level data will also be assessed with the national Area Deprivation Index (ADI) percentile, derived from each participant’s full address. Each SDOH factor will be scored 0 for protective or 1 for risk, determined by evidence-based ranges and then summed to calculate an SDOH Risk Index score (0 indicating no risk to 6 indicating high risk). This approach accounts for the additive burden of numerous SDOH variables [63]. The following SDOH factors will be coded as 1 for high risk: (1) education up to and including high school (beyond high school is protective [64]); (2) household income below US Federal Poverty guidelines (above is protective [65]); (3) no marital or partner is a risk (presence of a partner is protective [66]); (4) being unemployed, disabled, a student or homemaker is a risk (being employed or retired is protective [67]); (5) no medical insurance is a risk (having medical insurance is protective [68]); (6) ADI percentile >80% is a risk (≤80% is protective [69]).

We will also collect three measures at study start (visit 2) to explore their utility when examining symptom burden, adherence, and treatment outcomes. These measures include the Experiences of Discrimination (EOD) questionnaire, which is a self-report measure that assesses how often people feel they have experienced unfairness on the basis of race, ethnicity, gender, age, religion, physical appearance, sexual orientation, or other characteristics [70]. Scores range from 10-60, with higher scores indicating greater lifetime discrimination. Participants will also be asked to complete the Multidimensional Scale of Perceived Social Support questionnaire, which is a self-report measure that assesses an individual’s perceived level of social support with family, friends, and significant others [71]. Scores range from 0-72, with higher scores indicating a greater perceived level of social support. The third measure will be the Centers for Disease Control’s Social Vulnerability Index (SVI) derived from each participant’s full address. The SVI is a more recently available community-level variable and incorporates census variables to identify vulnerable communities within counties based on 15 factors organized within 4 themes: SES, household composition, ethnicity/race/language, and housing transportation [72]. Higher values indicate greater vulnerability.

### Additional Measures

At study start (visit 2), after eligibility is determined, participants will complete several questionnaires to characterize the sample. The Cambridge-Hopkins questionnaire [73] will assess for restless leg syndrome (RLS). For those who screen positive, the severity of RLS will be determined with the validated patient-report International Restless Leg Syndrome study group severity rating scale [74]. This scale assesses symptoms over the past 7 days, and the total score ranges from 0 (no symptoms) to 40 (very severe symptoms). Patients who do not screen positive will be assigned a severity score of 0. The severity score will be used as a moderator in the statistical analysis (see below). Participants will also be assessed for obstructive sleep apnea (OSA) with the STOP-BANG questionnaire [75]. This questionnaire assigns a point for each symptom or risk factor, and this score will also be used as a moderator in the statistical analysis. The Positive and Negative Affect Schedule (PANAS) questionnaire [76] will assess for trait positive and negative affect, and will be used to derive predisposing dispositional traits (protective or vulnerable) [77]. These traits will also be examined as a potential moderator in the statistical analyses. Finally, participants will also complete two brief questionnaires about their study expectations at visit 3, after they are informed of the condition they have been randomized to, and a study satisfaction questionnaire at visit 7.

Participants will wear the Fitbit Charge 5 or 6 on their non-dominant wrist to monitor activity and sleep. The Fitbit Charge uses accelerometry and cardiac autonomic signals to estimate sleep and has been compared to gold-standard polysomnography, demonstrating differentiation of sleep and wake states superior to US Food and Drug Administration cleared actigraphy [78]. We will export data from the Fitbit Charge in 30-second intervals using the Fitabase software. To augment the sleep data collected by the Fitbit device, sleep diaries will be completed, and participants will text a research email account to identify when they are trying to fall asleep (bedtime) and at their final wake time. Bedtime and rise time data through the time-stamped text messages will allow for confirmation of correct sleep onset and offset and calculation of total sleep time, wake after sleep onset, and sleep efficiency [79]. These objective sleep parameters will be primarily used to verify participant adherence to the morning light treatment and sleep timing stabilization schedule, but will also be examined for group differences. Participants will also track their daily use of caffeine, alcohol, medications, exercise, and psychotherapy on a daily event log.

### Safety Measures

At each weekly visit after the start of treatment (visits 4-7), participants will complete a self-reported measure of physical and emotional symptoms they have experienced in the past week using the Systematic Assessment for Treatment Emergent Events (SAFTEE) as we and others have used in previous light treatment trials [37,52,80]. Unblinded research staff will assess the severity of symptoms and their relevance to the study and assigned group, to ensure no significant negative effects are associated with the study participation. If severity meets a pre-determined threshold based on SAFTEE, the unblinded research staff will alert an unblinded study physician to further assess the participant’s safety and risk for an adverse event. In addition, at each weekly visit and 3-month follow-up, suicide risk will be assessed using the Columbia Suicide Severity Rating Scale (C-SSRS) [81], and during weekly visits, mood worsening will be examined with the Beck Depression Inventory (BDI) [82]. At least annually, an independent safety monitor will review the reported side effects and confirm it is safe to continue the study.

### Statistical Power and Analysis

We plan to enroll 390 individuals, aged 18 years or older, who meet the 2016 revised diagnostic criteria for fibromyalgia [45] and have at least mild fibromyalgia symptoms (FIQR score of ≥31) [48] (see Textbox 1). We aim for 309 participants with complete data (103 per each of the 3 groups), assuming 20% attrition during the 5-week study period. The sample size will provide 80% power to detect a difference in the standardized effect sizes (changes from baseline to post-treatment) of 0.47, 0.16, and 0.05 in FIQR in morning bright light treatment, sleep timing stabilization, and TAU groups, respectively, with a two-tailed 0.05 level 1-way ANOVA test. The effect size in the morning bright light treatment group is clinically meaningful and detectable based on our previous study [37]. The effect size in the sleep timing stabilization arm is 1/3 of that in the morning bright light group, and we conservatively estimated an effect size of 0.05 in the TAU group due to study participation. The study should also have adequate power to detect a binary outcome of clinically meaningful improvement (≥30% from baseline) or achieving a treatment response (≥16.8 decrease in FIQR) [83].

We will conduct an intention-to-treat analysis. We will report descriptive statistics of the data and visually explore the summary statistics by group at each visit. We will analyze both primary and secondary outcomes using a mixed-effects longitudinal data model with participants as random intercepts to account for between-participant variability. Predictors will include post-randomization time, two intervention group indicators with TAU as the referent group, and the interactions of time by group indicators. We will examine the extent and pattern of key missing outcomes data, and if missingness is greater than 15%, we will use logistic regression to assess baseline characteristics predictive of missingness postrandomization and will include those baseline characteristics as covariates in the final model. While FIQR scores will be examined as a continuous variable, we will also examine the proportion of participants who show clinical response (a binary outcome of clinically meaningful improvement (≥30% from baseline) or achieving a treatment response (≥16.8 decrease in FIQR [83]) and analyze the binary outcome variable using a generalized linear mixed model with logit link. We will also explore the severity of restless leg syndrome, obstructive sleep apnea, dispositional trait, and biological sex as potential moderators of any treatment effects.

## Results

The study was funded in July 2024. Data collection began in September 2024 and is projected to end in March 2029, with final analysis complete by June 2029. As of mid-July 2025, we have enrolled 55 participants.

## Discussion

This protocol paper describes a single-center 3-arm randomized controlled trial of morning bright light treatment versus sleep stabilization alone versus TAU in people with fibromyalgia. We hypothesize that (1) participants who receive morning bright light treatment will show greater improvements in function and pain, (2) that SDOH risk will influence baseline symptom burden, treatment adherence (engagement), and treatment response, and (3) that treatment response may be partially mediated by improvements in depressive symptoms and sleep, and a shift toward morningness. This study will provide further insight into the effect of morning bright light treatment on function, pain, depression, circadian timing, and sleep in people with fibromyalgia, and inform on its role as an adjunctive therapy for patients with active symptoms. In addition, the results will help reveal SDOH barriers to assist the future dissemination and implementation of morning bright light treatment in a variety of communities.

As pharmacological therapies for fibromyalgia offer only modest benefits [11-14] and result in a high rate of discontinuation due to adverse effects [11], newer national guidelines suggest that the initial approach to the treatment of fibromyalgia consists of nonpharmacological interventions [15-17]. Physical therapy/exercise and behavioral therapies such as CBT have demonstrated efficacy but can be difficult to access in terms of cost and availability [18-20], and engagement can be problematic [21]. In our earlier study [37], we found a reduction in the FIQR similar to reductions observed after weeks of exercise, and double that seen with weeks of CBT or the use of analgesics, as reported in a recent meta-analysis [38]. Furthermore, there is a good rationale for morning bright light treatment improving fibromyalgia symptoms, due to its recognized effects on mood [22,23], sleep [24], and circadian timing [27] (see Figure 1). Thus, morning bright light treatment has good potential as a readily accessible adjunctive approach to manage fibromyalgia symptoms, which can be self-administered with minimal side effects.

The strength of this study includes the randomized trial design with a control group, blinding of study staff directly involved in assessing study outcomes, and a well-described conceptual model of the potential mechanistic relationship between morning bright light treatment and study outcomes. In addition, we plan for a large sample size of 390 eligible participants for good statistical power, and the study will be entirely remote, which will assist the participation of eligible participants from a wide range of sociodemographic backgrounds across the United States. The feasibility of the planned approach is high given our team’s previous experience in conducting large remote clinical trials [84,85] and obtaining good treatment adherence and good-quality outcome data in our earlier pilot study (n=60) [37]. While the primary outcome measure, FIQR, is a self-report questionnaire, it is widely used in clinical trials for assessing changes in functional status and the global impact of symptoms in fibromyalgia [55]. The BPI has also been validated for fibromyalgia specifically [54,55]. We are using the Re-timer light therapy glasses because of the added convenience of enabling participants to be ambulatory during the morning light treatment, but given our earlier pilot work [35], we anticipate that our study results will also generalize to the use of bright light boxes. The anticipated study limitations include that the interventions are only 4 weeks long, although a study follow-up assessment 3 months after active treatment ends will help inform on longer-term treatment benefits of morning bright light treatment in people with fibromyalgia. We also note that, as a fully remote study, some degree of technological literacy is required of participants, such as knowing how to join an online Zoom call, and participants need to have internet access (Textbox 1).

This is the first National Institute of Health (NIH)–funded large randomized controlled trial of morning bright light treatment in fibromyalgia and has the potential to improve our understanding of the impact of morning bright light treatment on patients with nociplastic pain and fill a gap in adjunctive nonpharmacological fibromyalgia treatment. This study will also increase our understanding of the mechanistic relationships between mood, circadian timing, sleep, and fibromyalgia symptoms. If this large trial continues to support morning bright light treatment as efficacious for fibromyalgia, future research protocols can test the implementation and dissemination of morning bright light treatment for fibromyalgia and, potentially, other nociplastic pain conditions. At the conclusion of the trial, results will be disseminated via presentations, scientific publications, and self-help materials provided by the Chronic Pain and Fatigue Research Center at the University of Michigan. Summary data will be available on clinicaltrials.gov, and deidentified individual participant data will be available from the National Sleep Research Resource (see Data Availability statement).

## Abbreviations

ADI: Area Deprivation Index
BDI: Beck Depression Inventory
BPI: Brief Pain Inventory
CBT: cognitive behavioral therapy
C-SSRS: Columbia Suicide Severity Rating Scale
EOD: experiences of discrimination
FIQR: Fibromyalgia Impact Questionnaire–Revised
MEQ: Morningness-Eveningness Questionnaire
NIH: National Institutes of Health
OSA: obstructive sleep apnea
PANAS: positive and negative affect schedule
PHQ-9: Patient Health Questionnaire—9
PROMIS: Patient-Reported Outcomes Measurement Information System
REDCap: Research Electronic Data Capture
RLS: restless leg syndrome
SAFTEE: systematic assessment for treatment emergent events
SDOH: social determinants of health
SVI: Social Vulnerability Index
TAU: treatment as usual

## Data Availability

The datasets generated during this study will be available in the National Sleep Research Registry repository [86].
